# Deficit in hippocampal ripples does not preclude spatial memory formation in APP/PS1 mice

**DOI:** 10.1038/s41598-019-56582-w

**Published:** 2019-12-27

**Authors:** Bartosz Jura, Nathalie Macrez, Pierre Meyrand, Tiaza Bem

**Affiliations:** 1 Nałęcz Institute of Biocybernetics and Biomedical Engineering, Polish Academy of Sciences, 02-109 Warsaw, Poland; 2 Université de Bordeaux, UMR 5293, Institut des Maladies Neurodégénératives, 33000 Bordeaux, France; 3 CNRS, UMR 5293, Institut des Maladies Neurodégénératives, 33000 Bordeaux, France; 4 Present Address: INSERM, Neurocentre Magendie, U1215, University Bordeaux, 33-077 Bordeaux, France

**Keywords:** Alzheimer's disease, Neural circuits

## Abstract

General theory of declarative memory formation posits a cortical-hippocampal dialog during which hippocampal ripple oscillations support information transfer and long-term consolidation of hippocampus dependent memories. Brain dementia, as Alzheimer disease (AD), is accompanied by memory loss and inability to form new memories. A large body of work has shown variety of mechanisms acting at cellular and molecular levels which can putatively play an important role in the impairment of memory formation. However, far less is known about changes occurring at the network-level activity patterns that support memory processing. Using freely moving APP/PS1 mice, a model of AD, we undertook a study to unravel the alterations of the activity of hippocampal and cortical circuits during generation of ripples in the transgenic and wild-type mice undergoing encoding and consolidation of spatial information. We report that APP/PS1 animals are able to consolidate spatial memory despite a major deficit of hippocampal ripples occurrence rate and learning dependent dynamics. We propose that these impairments may be compensated by an increase of the occurrence of cortical ripples and reorganization of cortical-hippocampal interaction.

## Introduction

Despite considerable research on Alzheimer’s disease (AD) (from genes to behavior), there is still no cure and available treatments are only symptomatic. Effective rehabilitation strategies can rely only on a thorough understanding of the mechanisms underlying the cause of functional deficits and thus possible pathways to recovery. One of the symptoms of AD is the progressive impairment of memory. The cognitive impairments associated with AD are related to degenerative synaptic changes produced by the presence of soluble amyloid-β proteins (Aβs) in vulnerable brain regions^1,2^. Such accumulation of Aβ had been well described in transgenic mice which over express mutant human genes for amyloid precursor protein (APP) and presenilin1 (PS1) (APP/PS1 mice). The APP/PS1 mice gradually develop memory deficiencies which correlate with Aβs deposition^3^. Whereas basal synaptic transmission, long-term potentiation and long-term depression has been investigated at the cellular level in hippocampal slices *in vitro*^4–6^, studies on changes occurring at the circuits level during memory formation in freely moving APP/PS1 mice are still absent.

In the intact brain of rodents a large body of work has been focused on memory formation driven by hippocampal ripples, the high frequency network events generated during slow wave sleep (SWS) and consummatory behaviors^7–10^. Their major function is the transfer of information between hippocampus and cortex while other subcortical structures are silent^11^. Ripple oscillations are coordinated with cortical spindles and delta oscillations providing interactions between these structures^12,13^, whereas disturbing this dialogue impaired the memory formation^14,15^.

We therefore undertook a study to uravel the alterations of the activity of hippocampal and cortical circuits during generation of ripples in APP/PS1 mice undergoing encoding and consolidation of spatial information. To this end, we submitted mice to spatial recognition memory testing in a classical version of the 8 arm-maze discrimination task. We then explored ripple-associated interaction between cortex and hippocampus during the course of memory formation and its alteration due to AD pathology. In this study we report that APP/PS1 animals are still able to consolidate spatial memory despite a major deficit of hippocampal ripples which may be compensated by an increase of the occurrence of cortical ripples and ripple-to-ripple cortical-hippocampal interaction or by an increased number of trial-and-error events.

## Results

### Spatial memory consolidation is preserved in APP/PS1 mice

**Figure 1:**
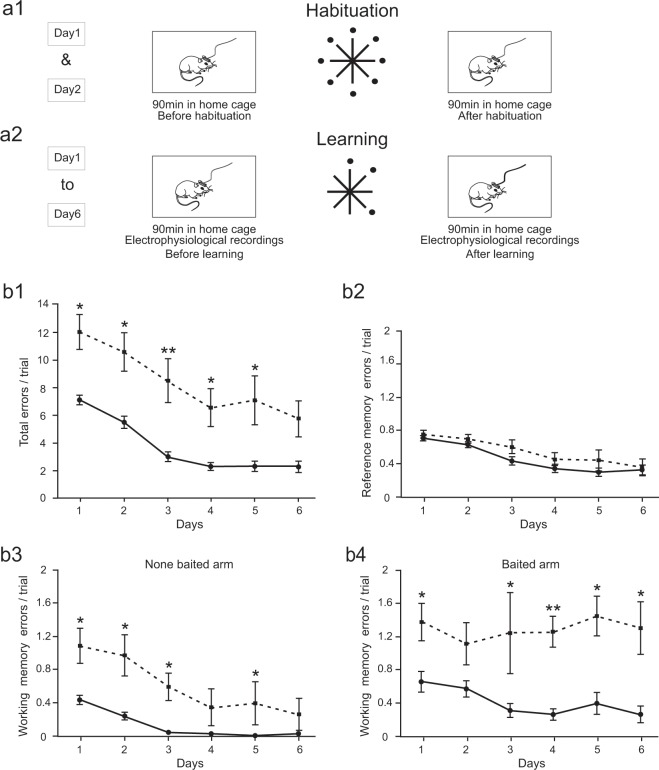
*APP-PS1* mice express impairment of working but not reference memory in 8-arm maze spatial memory test. (**a1-2)** Schematic representation of experimental paradigm. (**a1**) The animals were exposed to the experimental set up in their home cage and connected to the recording device before and after visiting the maze, where all the arms were baited. A daily learning session consisted in 6 trials repeated during 6 consecutive days. In each trial the animal had to find the position of 3 baited arms. Electrophysiological recording was performed in the home cage during 90 minutes before and after learning (**a2**). (**b1**) Evolution of total memory errors highlighting similar speed of learning in both groups and poorer performance in *APP-PS1* mice (P < 0.05 on days 1-5, Wilcoxon rank-sum with Bonferroni correction). (**b2)** Acquisition of reference memory is similar in both groups (P > 0.05 on days 1-6, Wilcoxon rank-sum with Bonferroni correction). (**b3)** Repetitive visits to none-baited arms were reduced during learning in WT but not APP/PS1 group (WT: N = 7, chi-sq = 29.89, P = 0.000; APP/PS1: N = 7, chi-sq = 7.55, P = 0.183, Friedman’s test), APP-PS1 expressing ~1 error /trial more than controls (P < 0.05 on days 1–3 and 5, Wilcoxon rank-sum with Bonferroni correction). (**b4)** The number of repetitive visits to baited arms remained constant in APP/PS1 mice and decreased in controls (WT: N = 7, chi-sq = 16.46, P = 0.006; APP/PS1: N = 7, chi-sq = 4.33, P = 0.503) which express less errors per trial (P < 0.05 on days 1 and 3–6, Wilcoxon rank-sum with Bonferroni correction).

To characterize the effect of APP/PS1 modification on spatial memory processing, APP/PS1 and wild-type littermate control (WT) mice were trained during 6 days to navigate in an 8-arm radial maze to learn the spatial position of three arms containing food rewards (Fig. 1a2). APP/PS1 mice performance remained consistently poorer than controls, showing ~5 errors per trial more during the course of learning (P < 0.05 on first 5 days, Wilcoxon rank-sum with Bonferroni correction) (Fig. 1b1). However, the slope of the learning curve was similar in both groups suggesting that APP/PS1 mice preserved some ability of learning the space.

To assess whether a specific kind of memory was altered in APP/PS1 animals we have compared the reference and working memory performance in each group. Surprisingly, the reference memory acquisition was not altered in APP/PS1 group, suggesting unimpaired spatial memory formation (Fig. 1b2). Indeed, APP/PS1 and control groups express similar number of reference memory errors per trial during the course of learning (P > 0.05 on days 1–6, Wilcoxon rank-sum with Bonferroni correction). By contrast, the working memory performance of APP/PS1 animals has shown impairment compared to controls. The number of repeated visits to non-baited arms decreases during learning in WT but not APP/PS1 group (WT: N = 7, chi-sq = 29.89, P = 0.000; APP/PS1: N = 7, chi-sq = 7.55, P = 0.183, Friedman’s test) and performance of APP/PS1 mice was poorer than controls (P < 0.05 on days 1–3 and 5, Wilcoxon rank-sum with Bonferroni correction). The impairment was also pronounced in re-visiting the baited arms (Fig. 1b4). By contrast to controls in APP/PS1 mice the number of repeated visits remained stable (WT: N = 7, chi-sq = 16.46, P = 0.006; N = 7, chi-sq = 4.33, P = 0.503, Friedman’s test) and consistently larger than in controls (P < 0.05 on days 1 and 3–6, Wilcoxon rank-sum with Bonferroni correction).

Altogether, these data indicate that although APP/PS1 animals expressed some deficits in performance of the spatial memory task, surprisingly their ability to learn the spatial position of baited arms was similar to the control group (Fig. 1b2).

### Hippocampal ripples are poorly expressed in APP/PS1 animals

**Figure 2:**
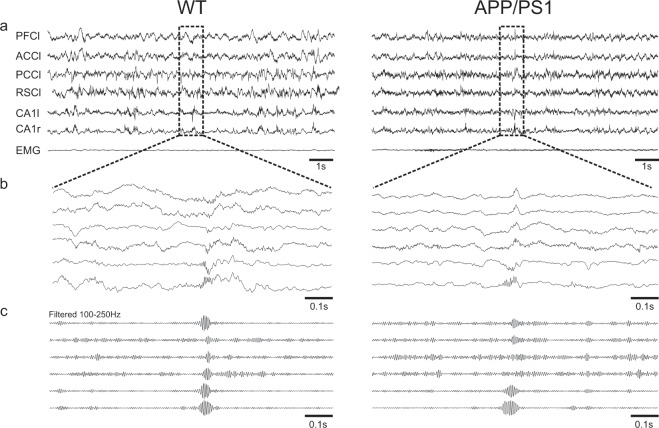
Representative example of co-occurring ripple-like oscillations generated in different cortical areas and hippocampal ripples in the wild-type and APP/PS1 animals. (**a)** Simultaneous recordings of LFPs from prefrontal cortex left (PFC*l*), anterior cingulate cortex left (ACC*l*), post cingulate cortex left (PCC*l*), retrosplenial cortex left (RSC*l*), dorsal hippocampus CA1 left and right (CA1*l*), (CA1*r*) and electromyogram of the neck muscle (EMG) during slow wave sleep (SWS). (**b,c)** Enlargement of 1 sec recordings of the LFPs showing co-occurring ripple oscillations in raw (**b**) and filtered (**c**) data.

In order to verify whether and how neural circuits that are involved in spatial memory processing were altered in APP/PS1 mice we recorded in freely moving animal the extracellular field potential in the left and right CA1 regions of the hippocampus along with left cortical regions of prefrontal cortex (PFC), anterior and posterior cingulate cortices (ACC and PCC respectively) and retrosplenial cortex (RSC). Moreover, to identify more accurately behavioral state of the animal (awake, slow wave sleep (SWS) and rapid eye movement sleep (REM)) the EMG of the neck muscle was recorded (Fig. 2). The recordings were performed in the home cage during 90 min immediately prior and after the learning sessions (see Fig. 1a2).

First, we compared occurrence of sleep episodes in both groups. Fractions of the total duration of SWS or REM sleep episodes within the time course of recording sessions were similar both before and after learning; before learning (APP/PS1: 0.53 ± 0.08, N = 7, WT: 0.59 ± 0.05, N = 7, P = 1, Wilcoxon rank-sum (SWS); APP/PS1: 0.089 ± 0.03, N = 7, WT: 0.097 ± 0.04, N = 7, P = 0.71, Wilcoxon rank-sum (REM)), after learning (APP/PS1: 0.46 ± 0.05, N = 7, WT: 0.40 ± 0.05, N = 7, P = 0.38, Wilcoxon rank-sum (SWS); APP/PS1: 0.077 ± 0.013, N = 7, WT: 0.053 ± 0.026, N = 7, P = 0.053, Wilcoxon rank-sum (REM)).

We then focused on the analysis of hippocampal ripples occurring during SWS. These ripples are known to be involved in spatial memory consolidation in post learning periods^14–19^. Occurrence rate, oscillation frequency, power and duration of ripples expressed in CA1 before and after the learning session during 6 days of training were calculated in both groups. No changes of these ripple features expressed either before or after the learning session were observed in the course of learning in both groups. (P > 0.05 for any ripple features before or after learning session, Friedman’s test,). Data from 6 days of recordings before learning session or after learning session were therefore combined for each animal.

**Figure 3:**
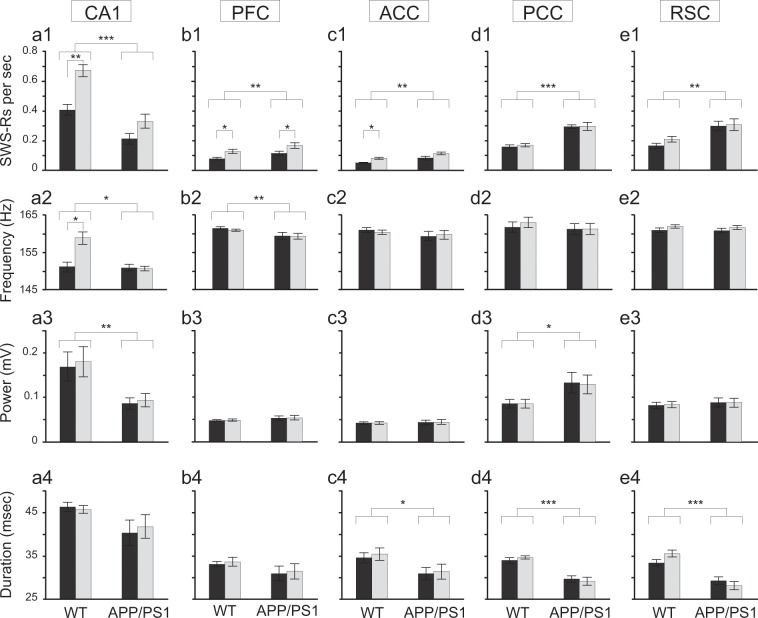
Impairment of hippocampal ripples and enhanced expression of cortical ripples during SWS in APP-PS1 mice. (**a1-4)**. CA1 ripples in *APP-PS1* group expressed lower occurrence rate (APP/PS1: N = 7, WT: N = 7, chi-sq = 17.23, P = 0.000, Friedman’s test), frequency (APP/PS1: N = 7, WT: N = 7, chi-sq = 4.39, P = 0.038, Friedman’s test) and power (APP/PS1: N = 7, WT: N = 7, chi-sq = 7.35, P = 0.007, Friedman’s test) than controls whereas ripple duration remained similar in both groups (APP/PS1: N = 7, WT: N = 7, chi-sq = 1.84, P = 0.17, Friedman’s test). Post-learning increase of ripple occurrence rate (WT: N = 7, P = 0.005, Wilcoxon rank-sum; APP/PS1: N = 7, P = 0.106, Wilcoxon rank-sum) and oscillation frequency (WT: N = 7, P = 0.014, Wilcoxon rank-sum; APP/PS1: N = 7, P = 0.91, Wilcoxon rank-sum) was significant only in control animals. (**b1-4,c1-4)**. Cortical ripple occurrence rate was significantly higher in APP/PS1 than control mice in PFC (APP/PS1: N = 7, WT: N = 7, chi-sq = 6.86, P = 0.009, Friedman’s test) and ACC (APP/PS1: N = 7, WT: N = 7, chi-sq = 9,44, P = 0.002, Friedman’s test). Post-learning increase of ripple occurrence was expressed in controls, both in PFC (N = 7, P = 0.011, Wilcoxon rank-sum) and ACC (N = 7, P = 0.002, Wilcoxon rank-sum) as well as in APP/PS1 animals in PFC (N = 7, P = 0.038, Wilcoxon rank-sum). APP/PS1 mice expressed lower ripple frequency in PFC (APP/PS1: N = 7, WT: N = 7, chi-sq = 6.86, P = 0.009, Friedman’s test) and shorter ripple duration in ACC (APP/PS1: N = 7, WT: N = 7, chi-sq = 5.10, P = 0.024, Friedman’s test). No significant differences between groups were found in ripple frequency in ACC (APP/PS1: N = 7, WT: N = 7, chi-sq = 0.008, P = 0.93, Friedman’s test), ripple power in ACC (APP/PS1: N = 7, WT: N = 7, chi-sq = 0.008, P = 0.93, Friedman’s test) and PFC (APP/PS1: N = 7, WT: N = 7, chi-sq = 1.38, P = 0.24, Friedman’s test) and ripple duration in PFC (APP/PS1: N = 7, WT: N = 7, chi-sq = 1.6, P = 0.206, Friedman’s test). (**d1-4,e1-4)**. Cortical ripples occurred more frequently in APP/PS1 animals, both in PCC (APP/PS1: N = 6, WT: N = 7, chi-sq = 16.61, P = 0.000, Friedman’s test) and RSC (APP/PS1: N = 7, WT: N = 7, chi-sq = 10.58, P = 0.001, Freedman test). PCC ripples were expressed with a higher power in APP/PS1 group (APP/PS1: N = 6, WT: N = 7, chi-sq = 5.12, P = 0.023, Friedman’s test). However, duration of cortical ripples was shorter in APP/PS1 animals both in PCC (APP/PS1: N = 6, WT: N = 7, chi-sq = 15.70, P = 0.000, Friedman’s test) and RSC (APP/PS1: N = 7, WT: N = 7, chi-sq = 16.53, P = 0.000, Friedman’s test). No significant differences between groups were found in oscillation frequency in PCC (APP/PS1: N = 6, WT: N = 7, chi-sq = 0.11, P = 0.73, Friedman’s test) and RSC (APP/PS1: N = 7, WT: N = 7, chi-sq = 0.13, P = 0.72, Friedman’s test), as well as in ripple power in RSC (APP/PS1: N = 7, WT: N = 7, chi-sq = 0.20, P = 0.65, Friedman’s test). No learning-dependent changes were found in PCC and RSC ripple properties. All box plot represent mean values before (dark) and after (light) learning, vertical bars represent standard errors.

Our analysis revealed major impairments of CA1 ripples in the APP/PS1 animals (Fig. 3a). APP/PS1 mice expressed lower ripple occurrence rate, oscillation frequency and power than the control group; occurrence rate (APP/PS1: 0.270/sec ± 0.041, N = 7, WT: 0.537/sec ± 0.036, N = 7, chi-sq = 17.23, P = 0.000, Friedman’s test), oscillation frequency (APP/PS1: 150.803 ± 0.725 Hz, N = 7, WT: 154.974 ± 1.469 Hz, N = 7, chi-sq = 4.39, P = 0.038, Friedman’s test), power (APP/PS1: 0.090 ± 0.014 mV, N = 7, WT: 0.174 ± 0.034 mV, N = 7, chi-sq = 4.39, P = 0.038, Friedman’s test). Moreover, it has been shown that in normal condition ripple occurrence rate increases after learning^20–23^. This was indeed observed in WT but not in APP/PS1 animals (WT: N = 7, 0.404/sec ± 0.036, n = 40701 (before), 0.670/sec ± 0.04, n = 46077 (after), P = 0.0046, Wilcoxon rank-sum; APP/PS1: N = 7, 0.210 ± 0.037, n = 13698 (before), 0.330/sec ± 0.047, n = 21018 (after), P = 0.106, Wilcoxon rank-sum) (Fig. 3a1, compare dark (before learning) and light (after learning) bars). In addition, WT group expressed a learning-induced increase of ripple frequency which was not present in APP/PS1 mice (WT: N = 7, 151.118 ± 1.390 Hz, (before), 158.830 ± 1.644 Hz (after), P = 0.014, Wilcoxon rank-sum; APP/PS1: N = 7, 150.912 ± 0.884 Hz (before), 150.695 ± 0.613 Hz (after), P = 0.91, Wilcoxon rank-sum) (Fig. 3a2, compare dark (before learning) and light (after learning) bars).

In summary, in APP/PS1 mice hippocampal ripples were much less expressed than in controls, with the occurrence rate and power diminished by ~50% and oscillation frequency diminished by ~10%. Moreover, whereas control mice expressed a post-learning increase of both ripple occurrence rate and oscillation frequency, no learning-induced changes of hippocampal ripples frequency were found in the APP/PS1 group. Although there was a trend toward increase of the occurrence rate also among APP/PS1 mice it did not reach statistical significance.

### Cortical ripples are more prevalent in APP/PS1 mice

It was recently demonstrated in rat that some cortical areas display ripple-like oscillations concurrent with hippocampal ripples that may participate in the cortical-hippocampal interaction during memory consolidation^24^. We observed such cortical ripples also in WT and APP/PS1 mice (Figs. 2, 3b–e).

Interestingly, by contrast to hippocampal ripples, cortical ripples were more abundant in APP/PS1 than in control mice in PFC (APP/PS1: 0.142/sec ± 0.015, N = 7, n = 11035 WT: 0.104/sec ± 0.010, N = 7, n = 6832 chi-sq = 6.86, P = 0.009, Friedman’s test), ACC (APP/PS1: 0.099/sec ± 0.010, N = 7, n = 6374 WT: 0.066/sec ± 0.005, N = 7, n = 3851 chi-sq = 9,43, P = 0.002, Friedman’s test), PCC (APP/PS1: 0.296/sec ± 0.019, N = 6, n = 37057 WT: 0.164/sec ± 0.004, N = 7, n = 13074 chi-sq = 16.61, P = 0.000, Friedman’s test) and RSC (APP/PS1: 0.303/sec ± 0.035, N = 7, n = 49376 WT: 0.187/sec ± 0.017, N = 7, n = 14985 chi-sq = 10.58, P = 0.001, Friedman’s test) (Fig. 3b1–e1). The power of cortical ripples was similar in both groups except for PCC ripples which were expressed with a higher power in APP/PS1 group (APP/PS1: 0.131 ± 0.022 mV, N = 6, WT: 0.086 ± 0.010 mV, N = 7, chi-sq = 5.12, P = 0.023, Friedman’s test) (Fig. 3d3). Also the frequency of cortical ripple oscillation was similar in the two groups (~160 Hz) with the except for PFC, where the frequency was found slightly higher in control than APP/PS1 animals (APP/PS1: 159.315 ± 0.88 Hz, N = 7, WT: 161.17 ± 0.329 Hz, N = 7, chi-sq = 6.86, P = 0.009, Friedman’s test) (Fig. 3b2–e2). However, cortical ripples were generally of shorter duration in APP/PS1 than control mice, namely in ACC (APP/PS1: 0.031 ± 0.002 s, N = 7, WT: 0.0350 =  ± 0.001 s, N = 7, chi-sq = 5.10, P = 0.024, Friedman’s test), PCC (APP/PS1: 0.029 ± 0.001 s, N = 6, WT: 0.034 ± 0.0004 s, N = 7, chi-sq = 15.70, P = 0.000, Friedman’s test) and RSC (APP/PS1: 0.029 ± 0.001 s, N = 7, WT: 0.034 ± 0.001 s, N = 7, chi-sq = 16.53, P = 0.000, Friedman’s test) (Fig. 3c4–e4). Importantly, learning-induced increase of cortical ripple occurrence rate was observed in the control group, namely in PFC and ACC: (PFC) (WT: N = 7, 0.079/sec ± 0.009, n = 3550 (before), 0.128/sec ± 0.013, n = 3282 (after), P = 0.022, Wilcoxon rank-sum; (ACC) (WT: N = 7, 0.050/sec ± 0.003, n = 2009 (before), 0.081/sec ± 0.007, n = 1842 (after), P = 0.004, Wilcoxon rank-sum). Also in the APP/PS1 animals post learning increase of PFC ripple rate was significant (N = 7, 0.116/sec ± 0.014, n = 5144 (before), 0.168/sec ± 0.018, n = 5891 (after), P = 0.038, Wilcoxon rank-sum) (Fig. 3b1,c1).

Abundance of hippocampal ripples and their learning related increase^20–23^ as well as a cortico-hippocampal dialog occurring during SWRs generation^12,13,18,25,26^ are important factors underlying spatial memory formation. However, as shown in Fig. 1b2, APP/PS1 animals expressed similar ability to learn the spatial position of baited arms as controls, despite a strong deficit in both the occurrence rate of hippocampal ripples and their learning related dynamics (Fig. 3a1). We further asked whether the larger prevalence of cortical ripples in APP/PS1 compared to control animals could compensate the deficit of hippocampal ripples leading to a more efficient cortical-hippocampal interaction.

### ACC and PFC ripples interact with hippocampal ripples only in APP/PS1 animals

A temporal association between cortical and hippocampal ripples was therefore analysed by measuring the rate of CA1 ripples co-occurring with cortical ripples within a window of 50 ms. Such a co-occurrence may result from a stochastic association between elements of two chains of events. Obviously, when number of events in one chain is higher within a given time window (as number of CA1 ripples after learning) then events in two chains will co-occur by chance more likely without any causal interaction between them. However, if causal relationships exist between hippocampal and cortical ripples, the co-occurrence should be different from the stochastic level and it would be interesting to see whether it changes after learning. We found that it was indeed the case.

**Figure 4:**
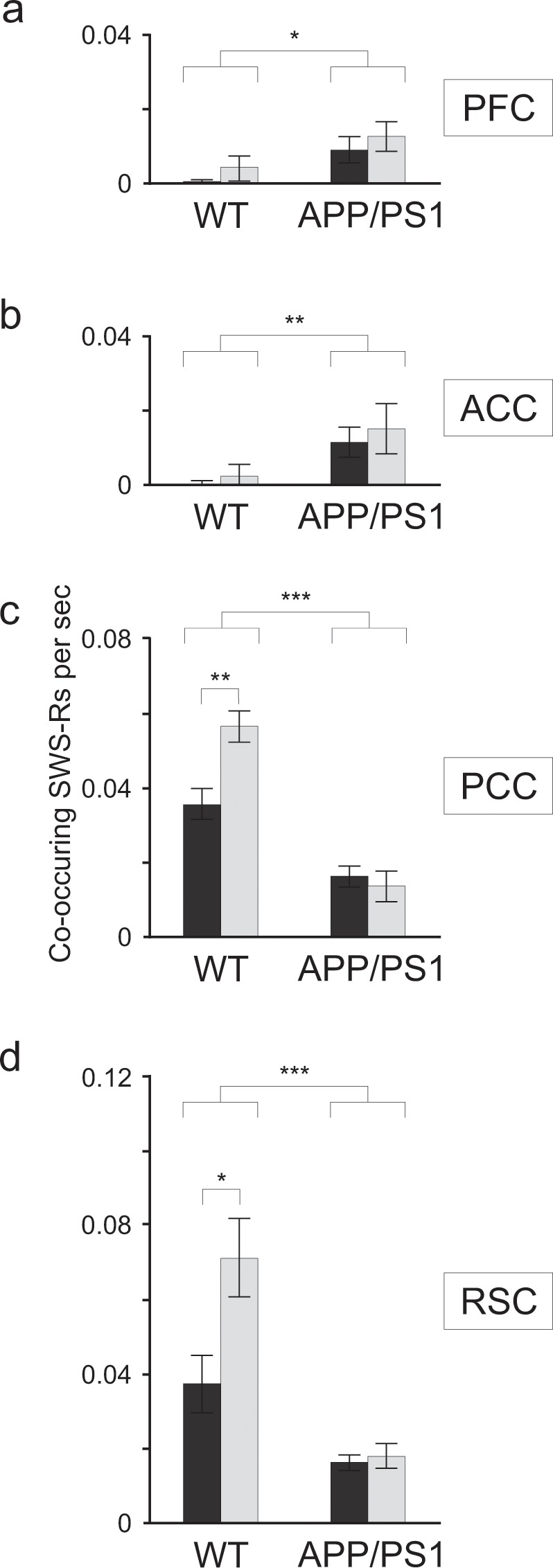
Co-occurrence of cortical and hippocampal ripples was altered in APP-PS1 mice. (**a,b)** In the control animals the rate of PCF and ACC ripples co-occurring with hippocampal ripples was not different from zero (P > 0.2, Wilcoxon signed rank), whereas in APP-PS1 animals the rate was significantly higher (ACC: N = 7, P = 0.004, Friedman’s test, PFC: N = 7, P = 0.011, Friedman’s test) but not learning dependent (P > 0.5, Wilcoxon rank-sum) **c,d**. In the WT group, the rate of PCC and RSC ripples co-occurring with CA1 ripples was significantly higher than in the APP-PS1 animals (N = 7, chi-sq = 13.13, P = 0.003, Friedman’s test (PCC); N = 7, chi-sq = 11.79, P = 0.001, Friedman’s test (RSC)). Moreover and in contrast to the APP-PS1 group the rate increased after learning (WT: N = 7, P = 0.008, Wilcoxon rank-sum; APP/PS1: N = 7, P = 0.93, Wilcoxon rank-sum (PCC); WT: N = 7, P = 0.026, Wilcoxon rank-sum; APP/PS1: N = 6, P = 0.804, Wilcoxon rank-sum (RSC)).

A “stochastic” co-occurrence was estimated by shuffling elements of temporal chain of hippocampal ripples (see Methods). Data illustrated in Fig. 4 show a “causal” co-occurrence obtained by subtracting a “stochastic” level from the co-occurrence measured in experiments. The occurrence of ACC and PFC ripples in control group was not causally related to hippocampal ripples (Fig. 4a,b). Indeed, in these animals the rate of “causal” co-occurence was completely negligible (comparison with 0, P > 0.2, Wilcoxon signed-rank). By contrast, in the APP/PS1 mice the rate of CA1 ripples co-occurring with ACC and PFC ripples was found to be significantly higher (ACC: 0.013/sec ± 0.005, N = 7, n = 1688, P = 0.004 Friedman’s test, PFC: 0.011/sec ± 0.004, N = 7, n = 1701, P = 0.011 Friedman’s test).

### PCC and RSC ripples interact with hippocampal ripples in controls but not APP/PS1 animals

The rate of CA1 ripples co-occurring with PCC and RSC ripples was substantially higher in the control group (APP/PS1: 0.015/sec ± 0.003, N = 6, n = 3169; WT: 0.046/sec ± 0.004, N = 7, n = 5010; chi-sq = 13.13, P = 0.003, Friedman’s test (PCC); APP/PS1: 0.017 ± 0.003, N = 6, n = 4093; WT: 0.054 ± 0.009, N = 7, n = 6305; chi-sq = 11.79, P = 0.001, Friedman’s test (RSC)). Moreover the rate of co-occurring CA1 ripples increased after learning in controls but not APP/PS1 animals (WT: N = 7, 0.036 ± 0.004, n = 2708 (before); 0.057 ± 0.004, n = 2302 (after); P = 0.008, Wilcoxon rank-sum; APP/PS1: N = 7, 0.016 ± 0.003, n = 1632 (before); 0.014 ± 0.004, n = 1537 (after); P = 0.93, Wilcoxon rank-sum (PCC); WT: N = 7, 0.037 ± 0.008, n = 3048 (before); 0.071 ± 0.011, n = 3257 (after); P = 0.026, Wilcoxon rank-sum; APP/PS1: N = 6, 0.016 ± 0.002, n = 2070 (before); 0.018 ± 0.003, n = 2023 (after); P = 0.804, Wilcoxon rank-sum (RSC)) (Fig. 4c,d).

In conclusion, cortical ripples were present in the control and APP/PS1 mice, expressing generally similar frequency, duration and amplitude (Fig. 3b–e). However, their relationships with hippocampal ripples were different in both groups. Whereas in controls strong interaction with hippocampal ripples was found only in PCC and RSC (Fig. 4c,d), APP/PS1 animals showed significant level of co-occurrence of hippocampal and cortical ripples in all cortical areas (Fig. 4a–d). Only in the control group the post-learning increase of co-occurring cortical ripples was found: namely in PCC and RSC. Since APP/PPS1 animals did not express any post-learning changes neither in the properties of hippocampal or cortical ripples nor in their mutual temporal relationships we further asked whether in this group some learning dependent relationships between cortex and CA1 may be realized in a form of cross-frequency modulation.

### Continuous comodulation of CA1 and cortical activity in the ripple frequency band during SWS

**Figure 5:**
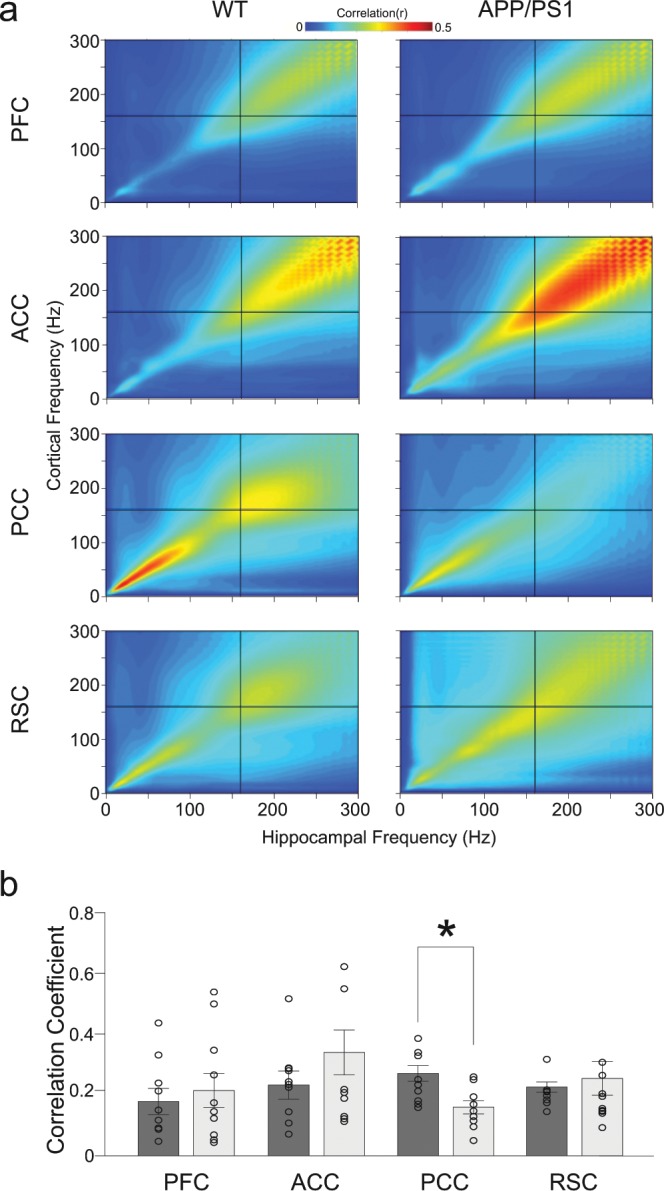
Comparison of cross-frequency power coupling between cortex and hippocampus in WT and APP/PS1 mice. (**a**) Comodulograms showing coupling between different cortical areas and CA1 during occurrence of cortical ripples, after learning session, averaged over 9 WT and 10 APP/PS1 animals. The cross indicates the mean frequency of cortical ripples. (**b**) Mean correlation coefficient between hippocampal and cortical activity in the cortical ripple frequency band (140–180 Hz). No statistical differences between WT (dark bars) and APP/PS1 group (light bars) were found except higher synchronization between CA1 and PCC in WT compared to APP/PS1 animals (P = 0.014, Wilcoxon rank-sum).

Cross-frequency power coupling between different cortical areas and CA1 was calculated in 0.5 sec time windows containing cortical ripples, centred in between ripple’s onset and offset, in the range of frequencies 0–300 Hz. First, we compared cross-frequency coupling after learning sessions in WT and APP/PS1 animals (Fig. 5a). In controls, but not in APP/PS1 animals a strong cortical-hipocampal comodulation in the ripple frequency range (150–250 Hz) was found in PCC (Fig. 5a). This finding was in accordance with previously demonstrated co-occurrence of CA1 and PCC ripples that was significantly higher in the WT than in APP/PS1 group (see Fig. 4c). Surprisingly, in both groups a strong comodulation in the ripple’s and higher frequency band (150–300 Hz) was found also in ACC (Fig. 5a), where the rate of co-occurring ripples was low or closes zero (Fig. 4b). Interestingly moreover, a similar level of comodulation in the frequency range 150–300 Hz was expressed apart from cortical or hippocampal ripples during SWS (data not shown), indicating synchronization of cortical and hippocampal activity occurring independently of ripple events. Comparison of the mean level of comodulation in the range of cortical ripples frequency (140–180 Hz) did not reveal significant differences between the two groups except for a higher synchronization of hippocampal and PCC activity in WT compared to APP/PS1 animals (P = 0.014, Wilcoxon rank-sum) (Fig. 5b). No learning dependent changes of the cortical -hippocampal comodulation were found neither in the WT nor in APP/PS1 group (data not shown).

## Discussion

Our results show that APP/PS1 are able to learn spatial reference memory task despite major impairment of hippocampal ripple features compared to their littermate control, suggesting an adaptive reconfiguration of neural circuits involved in spatial memory formation. We found that the capability to recognize spatial position of baited arms in the 8-arm maze is similar in APP/PS1 and control animals. Surprisingly, this ability of memory formation was accompanied by a substantial impairment of hippocampal ripple occurrence rate and learning dependent dynamics which are known to be crucially involved in spatial memory consolidation. Moreover, we found that the pattern of ripple related cortical-hippocampal interaction has been modified in APP/PS1 animals as compared to the control group. The APP/PS1 expressed more cortical ripples than controls with a temporal coordination with the hippocampal ripples that was widespread among all cortical sites studied, whereas in the control group such ripple-to-ripple interaction was restricted to fewer cortical areas. Finally, in both groups we found a strong co-modulation of CA1 and cortical activity in the ripple’s and higher frequency band (150–300 Hz) occurring during and apart from cortical and hippocampal ripples.

At the behavioural level the APP/PS1 animals show the impairment of the spatial memory. Nevertheless the reference memory has been not altered compared to the WT group. Using the same genotype, age and gender, another study showed that these mice exhibit spatial memory impairments^27^. Although animals were tested for spatial memory in both cases, it is difficult to compare the results. In Jankowsky *et al*. the animals were tested in the Morris water maze and then in the radial water maze. Such intensive pre-learning with different goals could explain the discrepancy between their results and ones presented in this study. Moreover, in our experiment the animals were food deprived and they were looking for the food rewards located always in the same places, whereas in Jankowsky *et al*. they were navigating searching for a safe place, the position of which was changing every day, therefore the level of stress and motivation could be quite different. Finally, although the reference memory measure in our task did not significantly change, we cannot exclude that impairment may exist in similar spatial tasks in other environments or measures at other time points.

A large body of research has shown that awake and sleep ripples play an important role in learning and memory formation^7,9,16–19^. Moreover, it has been demonstrated that disrupting hippocampal ripples during sleep impairs learning of spatial memory task^14,15^. Our results showed that in APP/PS1 animals the occurrence rate of ripples during SWS has been diminished by 50% compared to the control group (Fig. 3a1). This drastic drop was not leading to the deficit in spatial memory formation (Fig. 1b2). Moreover, the increase of the ripple rate that is typically observed after learning session^20–23^, was expressed only in the control group but not in APP/PS1 animals. In addition, whereas the controls expressed after-learning increase of the ripple intrinsic frequency, no changes have been noted in the APP/PS1 group. Despite these alterations of the APP/ PS1 ripple characteristics both groups show the same ability to encode and memorize the spatial position of baited arms.

All together these results may question the role of hippocampal ripples in spatial memory formation, at least in the spatial task used in our study. Interestingly, some recent study suggests that they are dispensable for the formation of stable space representation^28^. However, in this latter study, as well as in studies in which ripples generated during sleep were interrupted, all ripples have been truncated^14,15^, whereas in APP/PS1 animals ripples are still expressed although with the rate 50% lower than in the controls. Since hippocampal ripples can have different functions^9,29^ it cannot be excluded that mainly a specific type of ripples which do play the role in spatial memory formation was preserved in APP/PS1 animals. Nevertheless, the hippocampal network which generated these residual ripples was unable to respond to cognitive demands by increasing their occurrence after learning, suggesting that such transient increase of ripple rate which could provide a new, specific subset of ripples involved in processing of newly acquired hippocampal information is not necessary for spatial memory acquisition.

Another factor, beside the 50% of hippocampal ripples being still generated in APP/PS1 animals, which can contribute to their preserved ability of memorizing the spatial information, is the long lasting effect of ripple loss. It has been demonstrated that after injection of Aβ oligomer directly to hippocampus the animals lose both the ability to express an increase of ripple rate and capability to form spatial memory, tested two weeks after injection^23^. Similarly, testing of the memory performance was done shortly after ripple truncation^14,15^. By contrast, in the APP/PS1 mice the accumulation of Aβ oligomer and plaques (as well as presumable ripple deficits) occurs gradually over the course of few months^30,31,33^. Such long lasting changes may lead to adaptive responses, for instance a reconfiguration of circuits involved in memory formation, which could provide new functional pathways used to encode and store spatial information.

During SWS and consummatory behaviors hippocampal ripples drive the neocortex plasticity leading to stable memory formation. Among the various cortical-hippocampal interactions that have been already described^12,13,25,26,32^ a recent study have found a learning-dependent ripple – to – ripple communication between hippocampus and association cortices occurring during sleep in rat^24^. Our study confirms these findings in mice, in which ripple-like oscillations were found in PFC, PCC, ACC and RSC (Fig. 3b–d). Interestingly, the coupling between cortical and hippocampal ripples was organized differently in the two groups of animals: whereas in controls only PCC and RSC ripples showed temporal coordination with hippocampal ripples that increased after learning, in the APP/PS1 group the coupling was found in all recorded cortical areas suggesting a reconfiguration of cortical-hippocampal circuits (Fig. 4). However, in this latter case, no learning-dependent changes were expressed. Our analysis has also revealed in both groups a strong co-modulation of the cortical and hippocampal activity occurring in the 150–300 Hz frequency band, which has been independent of cortical or hippocampal ripple generation (Fig. 5). More experiments are needed to understand a functional role of this coupling which could be involved, as an alternative pathway, in a transfer of information between cortex and hippocampus.

Finally, another possibility to compensate the ripples deficits in the APP/PS1 group would be the increased number of repetitive visits during memory testing compared to controls (Fig. 1b4). Paradoxically, this enhanced re-visiting could be seen both as working memory impairment as well as a compensatory mechanism allowing for more trail-and-error experiences. Therefore, to memorize the position of baited arms the APP/PS1 animals, instead of repetitive reactivation of experience during sleep via ripples, could use awake reactivation of experience via repetitive visits in these arms.

## Material and Methods

### Animals and surgery

The mice used in the present study are double transgenic mice resulting from the crossing of 2 lines of commercial simple transgenic mice: APPswe, Tg2576 from Taconic maintained on a B6J background and PS1dE9 initially from Jax Lab and now maintained in Bordeaux facility on a C57BL6SJL background. Acute crossing of these two lines produces an accelerated mouse model of AD on a mix B6J/B6SJL background combining cognitive and amyloid pathologies starting as early as 4 months old as previously reported (Lagadec *et al*. 2012). This model has received ethical authorization # 3804 and 21377 from CEEA50, Bordeaux. All WT mice are littermates of APP/PS1 mice. All mice were heterozygous for each transgene. The genotypes were confirmed by polymerase chain reaction of tail biopsy. Data were collected from 10 APP/PS1^+^ and 9 WT females (8–9 months old). These animals were obtained in Bordeaux University animal facility and housed one per cage in a temperature (22 ± 1 °C) and humidity-controlled (50 ± 10%) conditions under an automatic 12 h light/dark cycle (lights on at 0700). Mice had *ad libitum* access to food and water prior the experimental procedure.

For the implantation of the multiple microelectrodes mice underwent stereotaxic surgery under deep isoflurane anesthesia. Microelectrodes, consisting of insulated tungsten wire (diameter 35 μm, California Fine Wires), were implanted using stereotaxic coordinates^34^ into: the Prefrontal Cortex (PFC) (AP: +2.0 mm, L: −0.36 mm, V: −1.6 mm), Anterior Cingular Cortex (ACC) (AP: +0.98 mm, L: −0.32 mm, V: −1.48 mm), Posterior Cingular Cortex (PCC) (AP: −2.0 mm, L: −0.3 mm, V: −0.8 mm), Retrosplenial Cortex (RSC) (AP: −3.0 mm, L: −0.5 mm, V: −0.8 mm) and CA1 region of left and right hippocampus (AP: −2.0 mm, L: −/+1.5 mm (left or right hemisphere), V: −1.05 mm). In all experiments, reference and ground electrodes were implanted into the cerebellum. The electromyogram (EMG) electrode was inserted into the neck muscles. All electrodes were welded to a connector attached to the skull with dental acrylic cement. After surgery animals were housed individually and had 3–4 weeks of recovery before the beginning of recordings and behavioral sessions.

Since brains of mice were used to perform histology and molecular biology analysis, we did not check the electrode location. However, the electrophysiological signature of the hippocampal ripples is well characterized and spatially restricted around the vicinity of the CA1 layer. Since we were able to see SWRs with bare eyes we were quite self-assured about electrodes placement. Examples of raw (black traces) and filtered (blue traces) hippocampal traces are shown in Suplementary Figures for WT (Figs. S2 and S3) and APP/PS1 animals (Figs. S4 and S5). Experimental procedures complied with official European Guidelines for the care and use of laboratory animals (directive 2010/63/UE) and were approved by the ethical committee of the University of Bordeaux (protocol A50120159 and A16323). Before starting spatial memory experiments mice were gradually food restricted to maintain their body weight at 85% of their *ad libitum* body weight throughout the experiments while access to water remained free. All procedures took place during the light cycle.

### Experimental procedure

To test the memory performance of mice an elevated eight-arm radial maze purchased from IMETRONIC (Pessac, France) was used (see Supplementary Fig. S1). This maze was composed of a central platform (30 cm in diameter) from which radiated eight identical arms (50 cm long and 11 cm wide). The entrance to each arm of the maze was controlled by automated sliding doors that could be controlled manually by an experimenter sitting in an adjacent room. Each arm was terminated by a small well in which food rewards were delivered. These rewards were small pellets of dehydrated milk. To avoid ‘olfactory cues’ the maze used was designed with automatic feeders where a reserve of pellets was located just under each arm and thus made a background smell that did not allow to discriminate between baited and non baited arms. The maze was located in a large room dimly illuminated, with various distal cues positioned on the walls. To provide a spatial hippocampal-dependent learning task the cues were not located in the continuation of the main axis of the baited arms, they were placed in between 2 arms and at a long distance from the extremity of the arms (1 - 2 meters). Prior to the start of each daily experimental procedure, food restricted mice were transported to the experimental room and remained undisturbed in their home cage for 30 min. Mice were familiarized with the radial maze and its environment during two days of habituation (Fig. 1a1). Each day, they first stayed in the home cage with the connector plugged to the recording system during 90 minutes. Then, they were disconnected and put into the maze where all arms were baited with food rewards. The daily session of habituation terminated when all eight baited arms were visited and at least one food reward was consumed. Finally, the animals were put back to their home cage where they stayed 90 minutes connected to the recording system. During learning the same procedure was used during 6 consecutive days, except that the food rewards were now distributed only in 3 arms of the maze: two adjacent arms and the third one separated by a non-baited arm (Fig. 1a2). Configuration of baited arms was randomly assigned to individual animals and kept constant during the course of learning. Each animal performed six trials per day. The trial was ended when all the rewards were eaten.

### Behavioral parameters

Total memory errors for each trial were defined as all visits to any arm of the maze that was not baited and repeated visits to arms that were previously baited in the ongoing trial. Reference memory errors were defined as the number of non-baited arms visited during trial expressed by one arm. Working memory errors were defined as the number of revisits (to baited or to non-baited arms) expresses by one arm.

### Data acquisition and data processing

During recording session, the mouse head connector was linked to amplifiers by a soft cable allowing free motions of the animal. Behavior was tracked with a video camera. Neurophysiological and EMG signals were acquired at 40 kHz on 128-channel Plexon system and stored on a PC for off-line analysis. Before further analysis, data were down-sampled to 1000 Hz using Matlab’s ‘decimate’ procedure. Identification of brain states was performed by visual inspection using several cues. First, *Sonic Vizualizer* was used to display and analyze the spectrograms. EMG was band-pass filtered to 250–350 Hz. Power spectra of delta (0.5–3 Hz) and theta (4–10 Hz) frequency band were calculated continuously. Second, *Neuroexplorer* was used to visualized brain activity in CA1 and cortical channels. Finally, brain states corresponding to awake, REM and slow wave sleep (SWS) states were manually scored by experimenter using EMG, spectrograms, delta/theta ratios as well as video-recording. REM states were identified as episodes of immobility with the presence of the high amplitude theta rhythm in CA1, absence of slow waves and no activity of the EMG. SWS states were identified as episodes of behavioral immobility, weak tonic EMG, low theta power and high delta power in CA1 and cortical channels, i.e. periods when amplitude of the delta band increased at least 1.5 times compared to awake state. Awake immobility were characterized, by contrast to SWS, by the presence of low amplitude high frequency activity in cortical channels.

**Figure 6:**
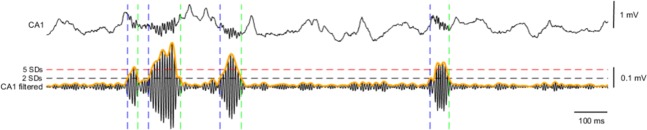
SWRs detection. Shown is wide-band trace of hippocampal CA1 signal and below the same trace filtered in the ripple frequency band (100–250 Hz). SWRs events are scored based on the relative instantaneous amplitude of the narrow band-filtered signal. Instantaneous amplitude is determined as envelope of the signal (indicated in orange), by taking amplitude of its Hilbert transform. Epochs in which value of the envelope exceeds mean + 2 SDs (black horizontal dashed line) if it reaches mean + 5 SDs (red horizontal dashed line) are considered SWRs events. The time points of the 2 SDs-crossing are taken as onset and offset points of SWRs.

Filtering of the signals was performed using Chebyshev Type II filter (order 4). Episodes of ripples were detected in signals filtered in the 100–250 Hz frequency band. Envelope of the narrow band-filtered signal, indicating its instantaneous amplitude, was calculated using the Hilbert transform. Envelopes were z-scored, using SD values calculated from the SWS bouts from the baseline recording sessions before learning. Ripple bouts were identified as epochs in which the envelope exceeded 2 SDs of the signal and reached 5 SDs, with the time points of the 2 SDs-crossing taken as onset and offset points of the ripple (Fig. 6). Episodes spaced less than 20 ms apart were merged and episodes longer than 100 ms were discarded. Ripple frequency was calculated using Hilbert transform of the signal, as time derivative of the instantaneous slope of the transformed signal. Ripple duration was calculated as an interval between 2 SDs-crossing points.

Comodulograms were calculated on 0.5 sec time windows centered on time points of ripple maximum amplitude. First, signal spectrograms were calculated using wavelet transform with Morlet wavelets and then Pearson’s correlation coefficient was calculated for all combinations of frequency bands between CA1 and every cortical channel.

### Statistical analyses

All statistical analyses were performed in Matlab (MathWorks). Results are expressed as mean ± SEM. Statistical tests used were non-parametric Wilcoxon’s rank sum test and Friedman’s test, as normality of data was not satisfied. Values of P < 0.05 were considered as significant. In case of multiple comparisons Bonferroni correction was applied. The data were collected from 19 adult female APP/PS1 and WT mice.

## Supplementary information

Supplementary Figures S1-S5
