# Supplementary material for: Deficit in hippocampal ripples does not preclude spatial memory formation in APP/PS1 mice
Source: Sci Rep. 2019 Dec 27;9:20129. doi: 10.1038/s41598-019-56582-w (PMC6934724; doi:10.1038/s41598-019-56582-w)
Supplement: Supplementary file 1 — Supplementary Figures S1-S5 [file 41598_2019_56582_MOESM1_ESM.pdf]

Deficit in hippocampal ripples does not preclude spatial memory formation in APP/PS1 mice

Bartosz Jura[1], Nathalie Macrez[2, 3], Pierre Meyrand[2,3 #], Tiaza Bem*[1#]

1. Nałęcz Institute of Biocybernetics and Biomedical Engineering, Polish Academy of Sciences, 02-109, Warsaw, Poland
2. Université de Bordeaux, UMR 5293, Institut des Maladies Neurodégénératives, 33000 Bordeaux, France
3. CNRS, UMR 5293, Institut des Maladies Neurodégénératives, 33000 Bordeaux, France
[#] These authors jointly supervised this work

Corresponding author: Tiaza Bem
Nałęcz Institute of Biocybernetics and Biomedical Engineering, Polish Academy of Sciences, 02-109, Warsaw, Poland
Email: tbem@ibib.waw.pl

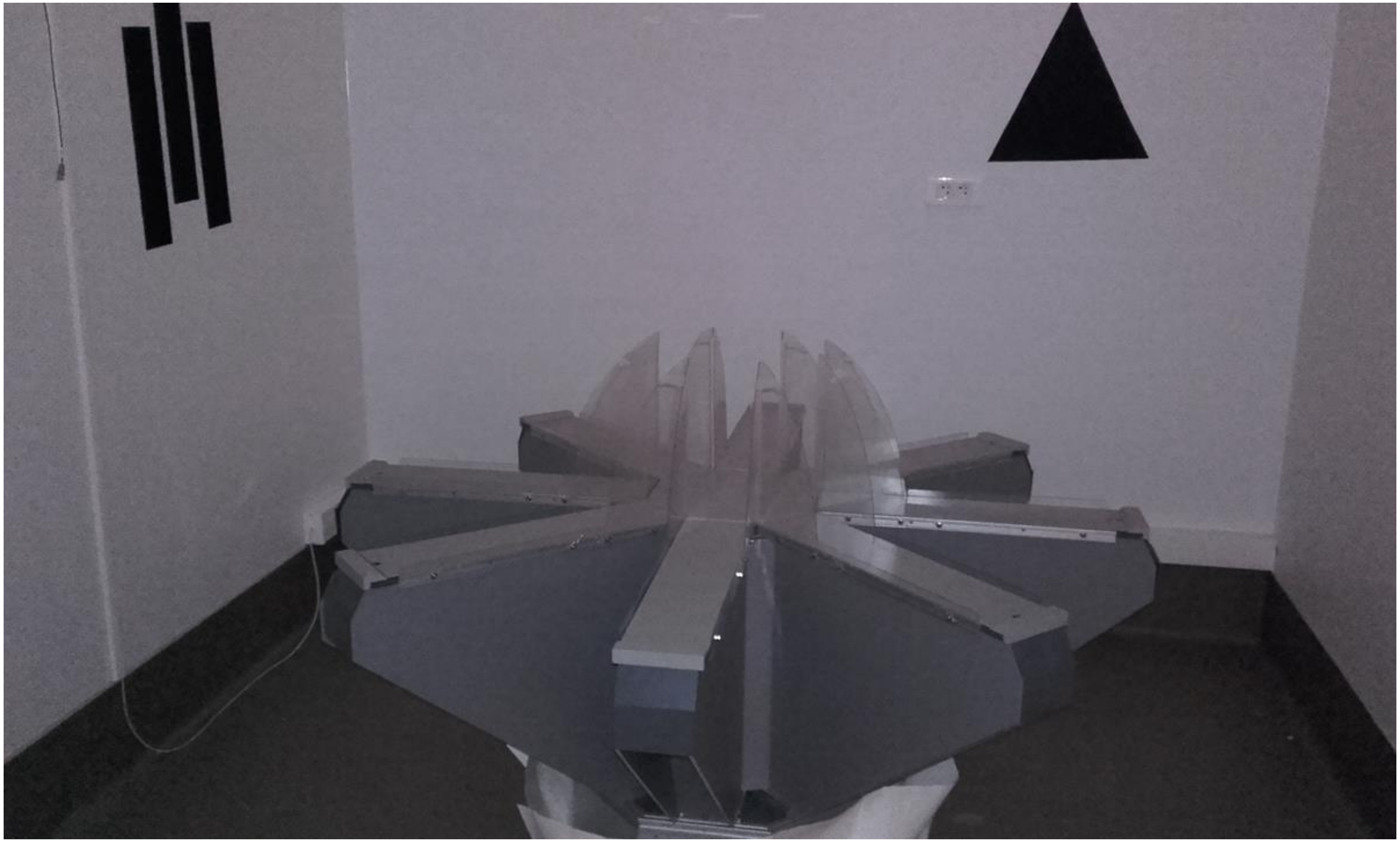

Supplementary Figure 1

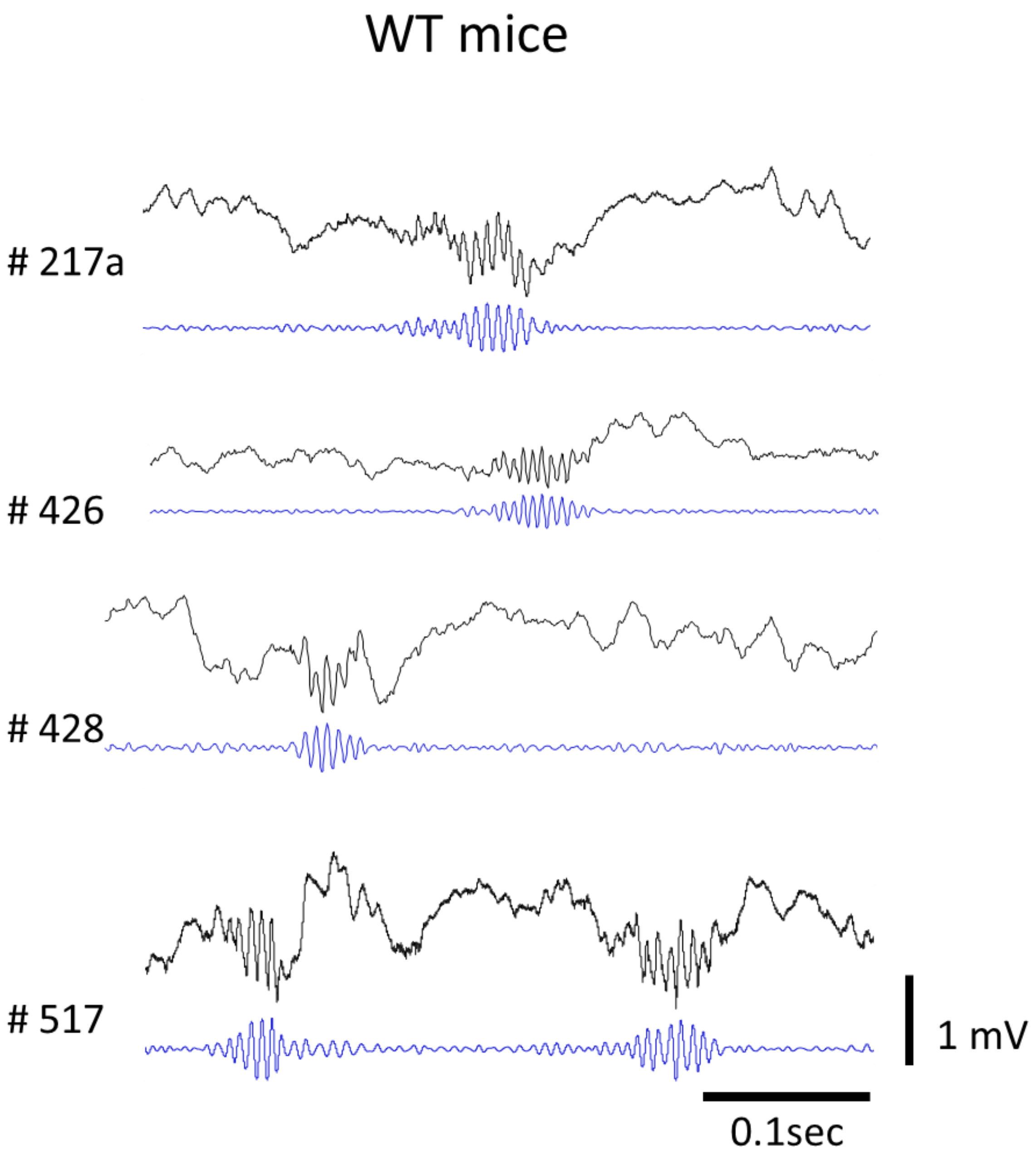

Supplementary Figure 2

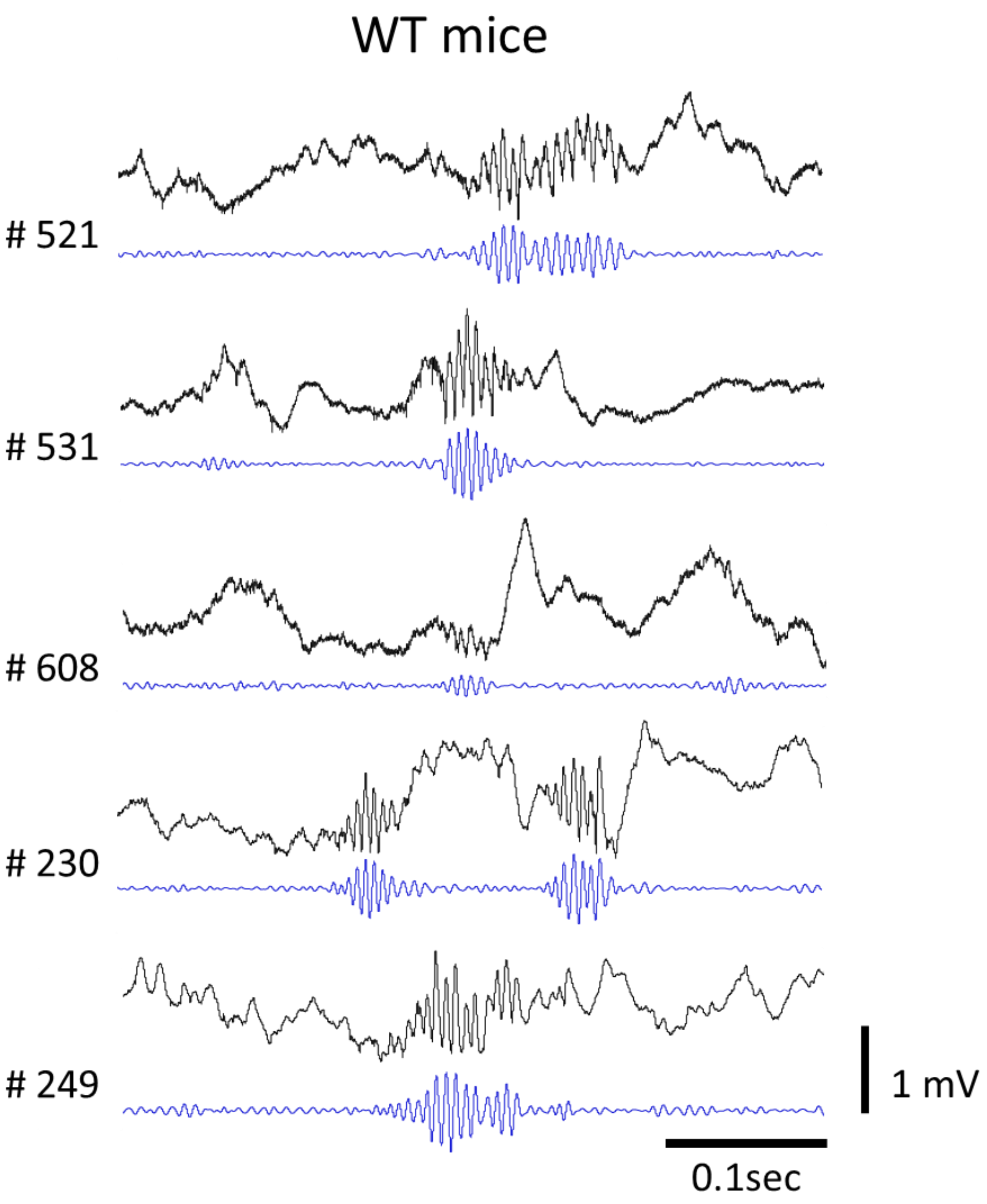

Supplementary Figure 3

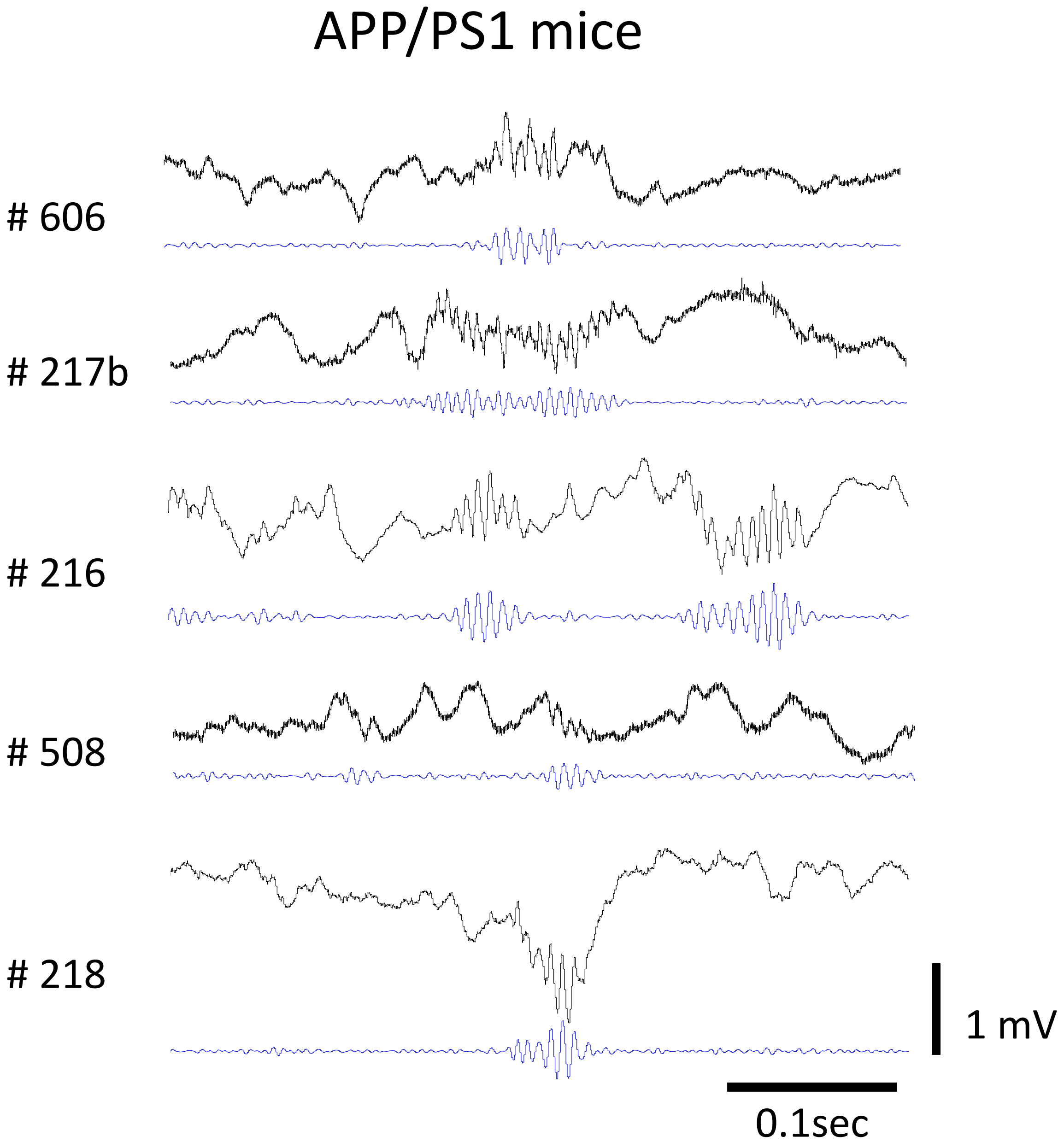

Supplementary Figure 4

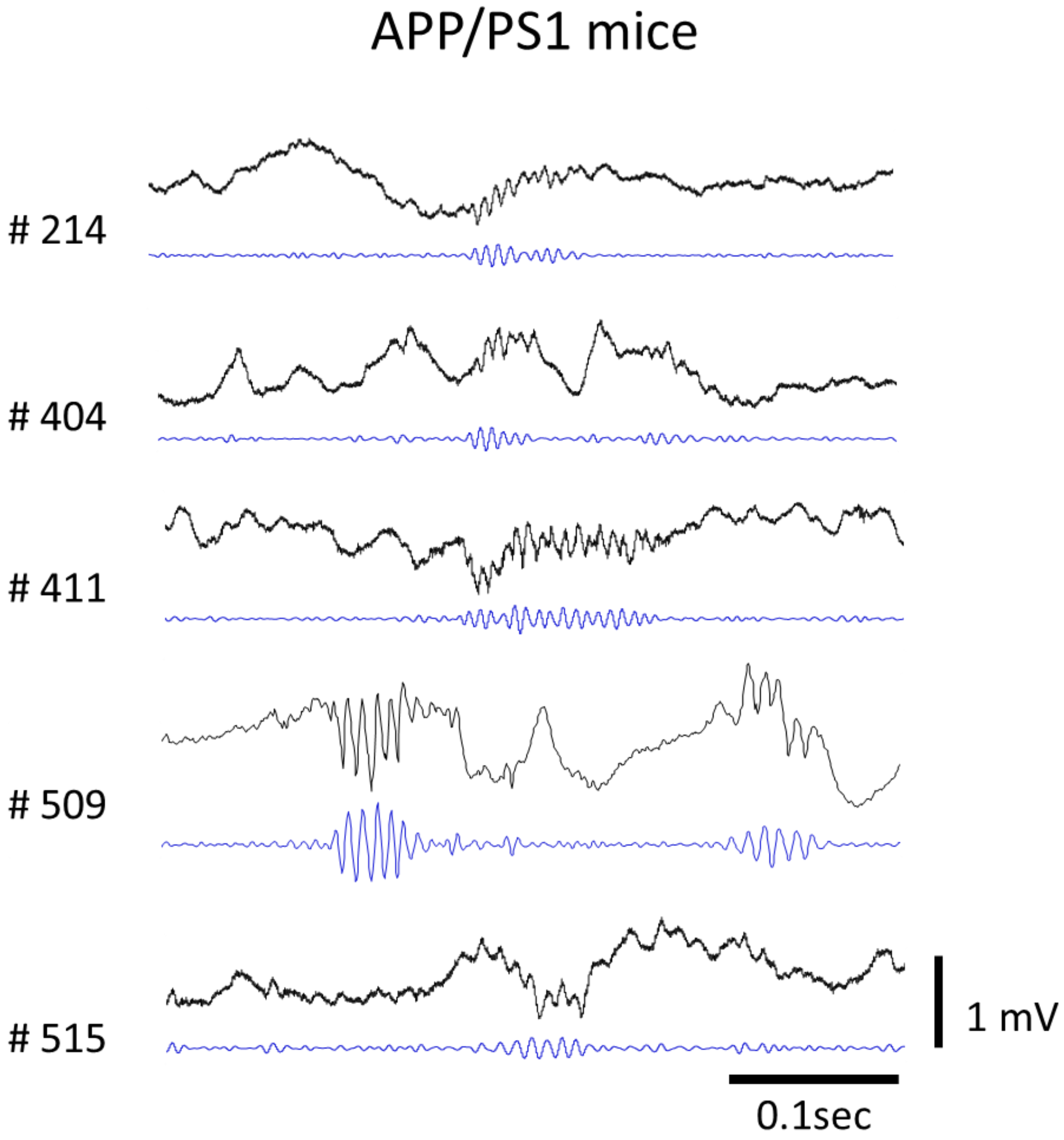

Supplementary Figure 5

Figure legends

Supplementary Figure 1: View of the behavioral experimental room. The 8-arm maze was placed at the center of the room, the cues were fixed on the walls (notice that the cues were neither close (> 100 cm from the extremity of the arms) nor located in the continuation of the main axis of the baited arms). During the course of the experiment each mouse had its own specific set of baited arms.

Supplementary Figure 2 - 3: Representative examples of SWRs in WT mice. Plot of raw traces and filtered (100 to 250 Hz) signals recorded from CA1 hippocampal region are represented by black and blue lines, respectively. Numbers on the left correspond to the identification of mice.

Supplementary Figure 4 - 5: Representative examples of SWRs in APP/PS1 mice. Plot of raw traces and filtered (100 to 250 Hz) signals recorded from CA1 hippocampal region are represented by black and blue lines, respectively. Numbers on the left correspond to the identification of mice.
